# Accuracy of classification of urinary Gram-stain findings by a computer-aided diagnosis app compared with microbiology specialists

**DOI:** 10.1099/jmm.0.002008

**Published:** 2025-04-23

**Authors:** Kei Yamamoto, Goh Ohji, Isao Miyatsuka, Kei Furui-Ebisawa, Ataru Moriya, Shogo Maeta, Hidetoshi Nomoto, Masami Kurokawa, Kenichiro Ohnuma, Mari Kusuki, Yukari Uemura, Norio Ohmagari

**Affiliations:** 1 Disease Control and Prevention Center, National Center for Global Health and Medicine, Tokyo, Japan; 2 Division of Infectious Diseases Therapeutics, Department of Microbiology and Infectious Diseases, Kobe University Graduate School of Medicine, Kobe, Japan; 3 Department of Clinical Laboratory, Kobe University Hospital, Kobe, Japan; 4 CarbGeM Inc., Tokyo, Japan; 5 Department of Clinical Laboratory, National Center for Global Health and Medicine, Tokyo, Japan; 6 Department of Clinical Laboratory, Ibaraki Higashi National Hospital, Ibaraki, Japan; 7 Center for Clinical Sciences, National Center for Global Health and Medicine, Tokyo, Japan

**Keywords:** artificial intelligence, computer-aided diagnosis, Gram staining, non-inferiority trial, urinary tract infection (UTI)

## Abstract

**Introduction.** Timely and accurate diagnosis of bacterial infections enables early administration of appropriate antimicrobial treatment and improved outcomes.

**Hypothesis/Gap Statement.** The accuracy of computer-aided diagnosis (CAD) for identifying organisms on urine Gram stains has not been compared with that of microbiology specialists (MS).

**Aim.** To compare the interpretation of urine Gram-stain results by MS and a CAD app designed using artificial intelligence.

**Methodology.** Urine specimens from patients with urinary tract infections were used and collected at two tertiary hospitals between 1 April and 31 December 2022. Using non-inferiority analysis to assess whether CAD was non-inferior to expert interpretation, CAD-predicted microscopic findings of the Gram-stained slide generated from iPhone camera images from two hospitals were compared with those from ten MS. A total of 153 images were taken from each hospital, and CAD interpreted a total of 306. The primary endpoint was the prediction accuracy based on the morphology of the Gram-stained bacteria.

**Results.** The accuracy (95% confidence interval) of MS and CAD predictions was 83.0% (81.6%–84.3%) and 87.9% (83.7%–91.3%), respectively, with a difference of –4.93% (–8.43% to –0.62%) indicating non-inferiority of CAD.

**Conclusion.** CAD was non-inferior to MS predictions for identifying Gram-stained pathogens; therefore, CAD was suggested to have the potential for guiding empirical antibiotic selection in patients with urinary tract infections.

## Data Availability

All data associated with this study are presented in the main text or appendix and are available from the authors upon reasonable request. Individual-level, de-identified participant data are not available for publication.

## Introduction

In individuals with bacterial infections, early administration of appropriate empirical antibiotics can improve outcomes [1,2]. However, traditional culture for bacteria requires time to determine the causative organism. Multiplex nucleic acid amplification tests and matrix-assisted laser desorption/ionization time-of-flight mass spectrometry can shorten the turnaround time, contributing to antimicrobial stewardship with appropriate support [3]. Although new point-of-care tests for urinary tract infections (UTIs) that use urine specimens directly are under development [4,5], such testing methods are not yet widely available. Moreover, the expense of installing and maintaining novel testing devices is a barrier to adopting new testing methods. Although the choice of empirical antibiotics is based on treatment guidelines, patient risk profile and symptoms, point-of-care Gram staining (PCGS) is also useful for informing empirical antibiotic selection.

In cases of UTI, urine Gram staining can contribute to diagnosis [6,7] and empirical antibiotic selection, thereby reducing broad-spectrum antibiotic use and the cost of antibiotics compared with guideline-based selection [8]. Urine specimens are relatively easy to collect, and although specimen quality evaluation is not required, proficiency is required to read and interpret the Gram-stain findings accurately [9,10]. Most facilities in Japan (62%) do not have clinical technologists who can perform Gram staining of urine specimens after hours [11]. Additionally, Gram-stain interpretation by non-specialists is significantly less accurate than that of microbiology specialists (MS) [12].

We developed an inexpensive computer-aided diagnosis (CAD) system using artificial intelligence (AI) to use as a tool to assist with bacterial identification and the selection of an appropriate initial antibiotic for treating UTI in settings in which 24 h Gram-stain interpretation by MS is not possible. We then conducted a non-inferiority study to investigate whether the accuracy of CAD interpretation of Gram-stain findings had similar accuracy to that of MS interpretation.

## Methods

### Study design

This retrospective observational study used residual clinical specimens from two tertiary care hospitals (the National Center for Global Health and Medicine [NCGM] and Kobe University Hospital [KUH]). The primary analysis was a non-inferiority test of CAD interpretation compared with the interpretation by 20 MS working in the two hospitals. The MS included physicians and clinical laboratory technicians who were infectious disease specialists certified by the Japanese Association of Infectious Diseases and technologists in microbiology certified by the College of Laboratory Medicine in Japan or by Medical Technologists in Clinical Microbiology.

The study was approved by the Institutional Review Board of the NCGM (NCGM-S-004480–02). Since residual clinical specimens were used retrospectively, the requirement for informed consent was waived.

### AI model building

A total of 1,350 urine specimens were collected for AI model building from existing anonymized NCGM and KUH registries between 1 February 2020 and 31 March 2022, and 1 January 2023 and 30 March 2023 (Table S1, available in the online Supplementary Material).

We used Python as the language and PyTorch (https://pytorch.org/) along with its wrapper PyTorch Lightning (https://lightning.ai/pytorch-lightning) as the deep-learning framework to construct the AI model. A total of 13,901 images (from 1,350 slides) were used to build the AI model. Details of the construction of the AI model are described in Appendix S1.

### Inclusion criteria and data collection

Gram-stained slides of urine specimens sampled between 1 April 2022 and 30 June, 2022, at NCGM and 1 April 2022 and 31 December 2022, at KUH were used to test the CAD system. Gram staining was performed at both sites using the Bartholomew and Mittwer (B and M) method [13]. Clinical data were collected on the sampling date, specimen type, Gram-stain findings and species identification results.

**Fig. 1.**
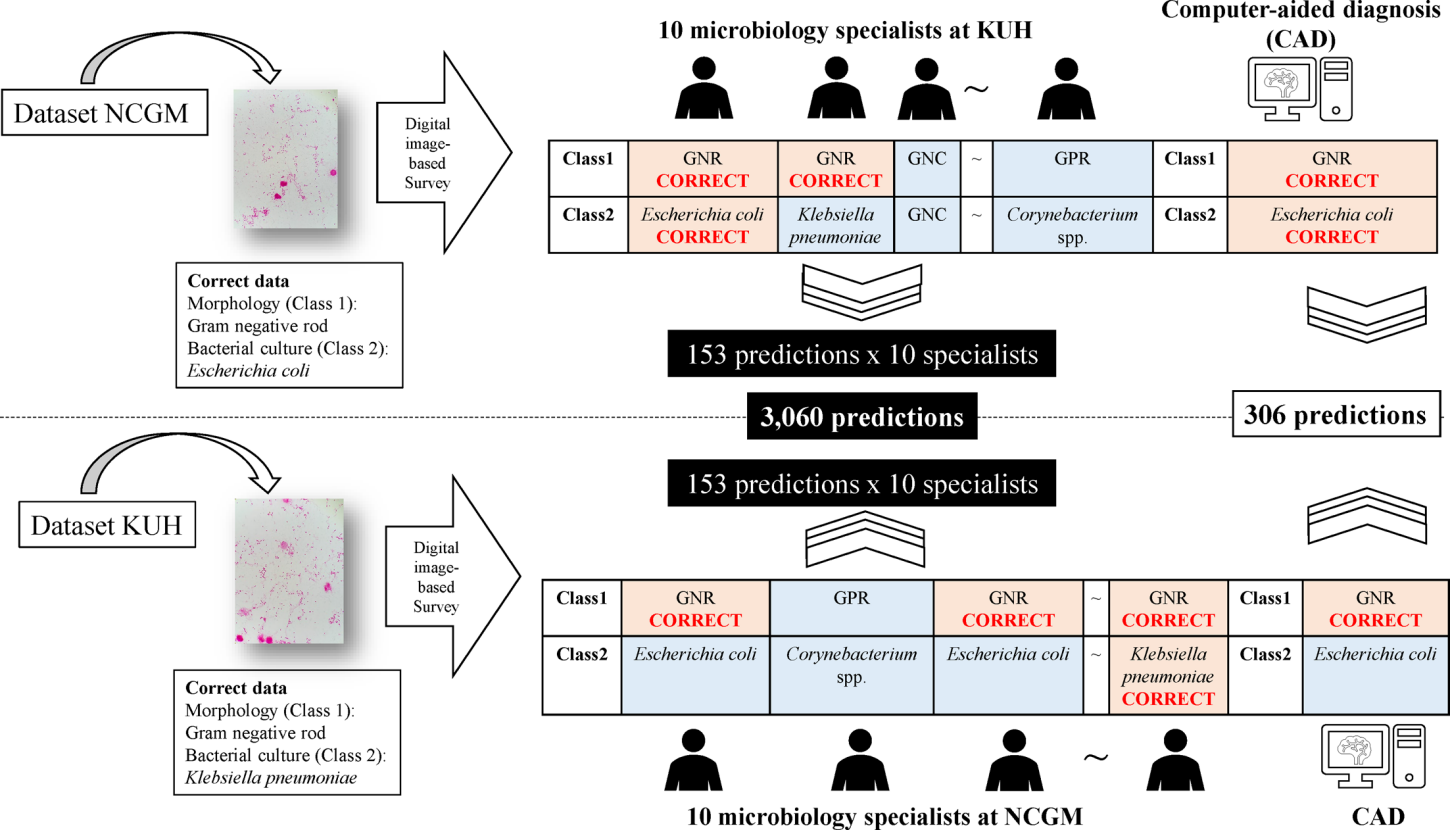
Method of reading Gram-stain microscopic images from the created image data set. Examples of predictions are shown. MS and CAD determine the shape of the bacteria (Class 1) and the general type of bacteria (Class 2) from a single digital image in a blinded state. Red cells indicate that the prediction was correct, and blue cells indicate that the prediction was incorrect. A total of 3,060 predictions were made, as 10 MS at each facility interpreted 153 images, and the CAD system made one prediction per image, making a total of 306 predictions. GNC, Gram-negative cocci; GNR, Gram-negative rod; GPR, Gram-positive rod; KUH, Kobe University Hospital; NCGM, National Center for Global Health and Medicine.

**Fig. 2.**
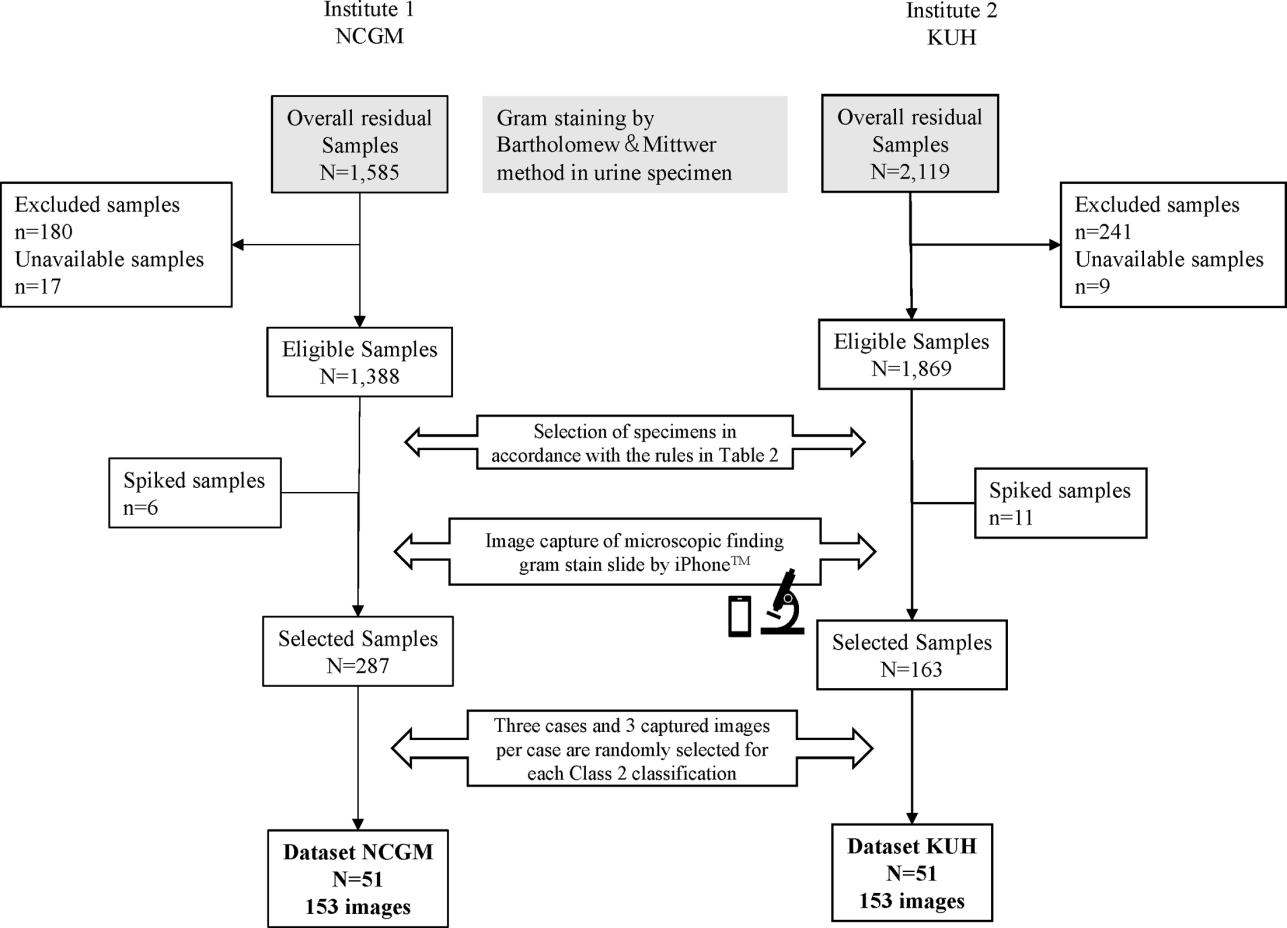
Study flow. This shows the flow of specimens up to the creation of the datasets used for analysis. KUH, Kobe University Hospital; NCGM, National Center for Global Health and Medicine.

Samples with both Gram-stain results and species identification (confirmed by bacterial culture) were included. Samples were excluded if a single organism was observed on microscopy, but more than one organism was identified on bacterial culture (e.g. Gram-negative rod [GNR] was observed by microscopy, but *Klebsiella pneumoniae* and *Escherichia coli* were isolated). Because the correct result for single microscopic images was based on the results of bacterial culture, it was not possible to determine the correct result when two or more types of bacteria were isolated. Samples were also excluded if the identified organisms differed (e.g. Gram-positive rod [GPR] observed by microscopy and *E. coli* was isolated) because if the microscopic findings and bacterial culture results differ, the possibility of culture contamination is high. Since the criteria for semi-quantitative culture may differ depending on the bacterial species, the results of semi-quantitative culture were not collected. The images used as training data did not overlap with the experimental images. The Gram-stained microscopic images were interpreted according to the Gram-staining classification shown in Table 1, with both the MS and CAD blinded to the bacterial morphology, Class 1, Class 2 and species (Fig. 1). Table 2 shows the number of images captured according to the number of samples by class. For classifications with no more than three samples, spiked samples generated using the American Type Culture Collection (ATCC) standard strain were added to create a total of at least three samples. The procedure for preparing the spiked specimens using ATCC standard strains is described in Appendix S2. In addition, ten images were captured per sample from 15 to 50 samples randomly selected among samples with undetected bacteria during microscopy (labelled as ‘none’), and samples with two or more bacteria with different morphology (e.g. not applicable to cases where Gram-positive cocci [GPC] clusters and chains were mixed but applicable where GPC and Gram-negative cocci [GNC] were mixed) were labelled as ‘polymicrobial’. From these samples, three were randomly selected in each species (Class 2) category, and three image files per sample were included in the study (Fig. 2).

**Table 1.** Gram-staining classification

Code	Class 1	Class 2
U-01	Yeast	*Candidaspp*.
U-02	GPC	GPC cluster
U-03	GPC	*Enterococcus faecalis*
U-04	GPC	*Enterococcus faecium*
U-05	GPC	*Streptococcus agalactiae*
U-06	GPC	Other GPC
U-07	GPR	*Corynebacteriumspp*.
U-08	GNR	*Enterobacter cloacae*
U-09	GNR	*Escherichia coli*
U-10	GNR	*Klebsiella oxytoca*
U-11	GNR	*Klebsiella pneumoniae*
U-12	GNR	Other GNR *Enterobacteriaceae*
U-13	GNR	*Pseudomonas aeruginosa*
U-14	GNR	Other GNR glucose non-fermenting bacteria
U-15	GNC	GNC
Poly	Polymicrobial	Polymicrobial
None	None	None

GNC, Gram-negative cocci; GNR, Gram-negative rod; GPC, Gram-positive cocci; GPR, Gram-positive rod.

**Table 2.** Number of target specimens extracted and images taken

Actual no. of samples from each institute	No. of samples included	No. of images per sample
0–4	3*	12
5–9	5	7
10–14	10	4
15–19	15	3
≥20	20	2

a*When the number of specimens available was <3, specimens were supplemented with spiked specimens to increase the number to 3.

### Device and interpretation

The imaging device was an iPhone (Apple, Cupertino, CA, USA; NCGM: iPhone Xs, KUH: iPhone 12) with a rear camera pixel count of at least 12 megapixels. Images were captured using the standard camera application (1.9×optical zoom) with an attachment, NexyZ universal smartphone adapter (Celestron, Torrance, CA, USA), to an optical microscope to focus the image from the microscope eyepiece. For the optical microscopes, at NCGM, BX53 with discussion microscope (Olympus, Tokyo, Japan) and, at KUH, CX41LF (Olympus, Tokyo, Japan) were used.

For the CAD used in this study, deep learning with supervised data generated from Gram-stained slides was collected from KUH and NCGM outside this study period and had been registered.

The image datasets were created at the two facilities and sent to different facilities in anonymized form for interpretation. Ten MS from each facility interpreted the findings using a PC display. After interpretation by the MS was completed, the same image dataset was sent to CarbGeM’s laboratory for interpretation by CAD. After CAD interpretation was completed, the MS and CAD interpretation results were sent to each facility where the dataset was created, and the results were checked against the correct data.

### Outcome

The primary outcome was accuracy, and results were considered accurate when the predicted data matched the correct data. The macro-average sensitivity, recall, precision and F1 score (harmonic mean of recall and precision) for Classes 1 and 2 were calculated as secondary outcomes. Recall, precision and the F1 score were calculated using the following equations [14], :


$$Accuracy=\frac{Truepositive+Truenegative}{All}$$

$$Recall=\frac{Truepositive}{Truepositive+Falsenegative}$$

$$Precision=\frac{Truepositive}{Truepositive+Falsepositive}$$

$$F1score=２\times \frac{Precision\times Recall}{Precision+Recall}$$

‘Recall’ is synonymous with sensitivity. It evaluates the true value that is missed due to the solution presented by CAD, whereas ‘precision’ evaluates the correctness of the solution presented. Both are important indicators for diagnostic devices, but the ‘F1 score’ is an indicator that evaluates the diagnostic performance by taking the harmonic mean of the values of these two indicators [14]. The agreement between correct and incorrect answers (positive and negative agreement) for MS and CAD was also evaluated as a secondary outcome.

### Sample size calculation

PASS 2023 (NCSS, LLC, Kaysville, UT, USA) was used for the sample size calculation. Based on the results of an unpublished pilot study that showed accuracies of MS and CAD in discriminating bacterial morphology (excluding the none and polymicrobial categories) of 96.4% and 95.7%, respectively, the target sample size was calculated to be 306 images in the dataset, 306 predictions by CAD and 3,060 predictions by MS, which was analysed based on a 5% non-inferiority margin and one-sided alpha error of 2.5%. Based on this sample size, the power was approximately 83%.

### Statistical analysis

Accuracy was expressed as point estimates with 95% confidence intervals (CI). Non-inferiority was analysed using Farrington and Manning analysis at a significance level of 5%. The agreement between correct and incorrect answers per image for each CAD and MS was evaluated using Cohen’s kappa coefficient and Gwet’s first-order agreement coefficient (AC1) statistics. Inter-rater reliability was evaluated using the Fleiss kappa coefficient and Gwet’s AC1 statistics. The kappa coefficient and the AC1 statistic were evaluated according to the strength of agreement of the kappa coefficient, using the following criteria: <0.0 poor, 0.00–0.20 slight, 0.21–0.40 fair, 0.41–0.60 moderate, 0.61–0.80 substantial and 0.81–1.00 almost perfect [15]. R version 4.3.0 (R Foundation for Statistics. Computing, Vienna, Austria) was used for statistical analysis.

## Results

Of the 1,388 eligible clinical samples from NCGM and 1,869 eligible samples from KUH, 253 and 152 samples, respectively, were included (Fig. 2, Tables S2 and S3). Groups of 6 (3 each of GNC and other GNR glucose non-fermenting bacteria) and 11 spiked samples (3 GNC, 3 other GNR glucose non-fermenting bacteria, 2 *Enterococcus faecium*, 1 other GPC and 2 *Klebsiella oxytoca*) were included in the NCGM and KUH datasets, respectively (Table S4). The MS group included 9 physicians and 11 laboratory technicians.

### Class 1 (morphological type)

**Fig. 3.**
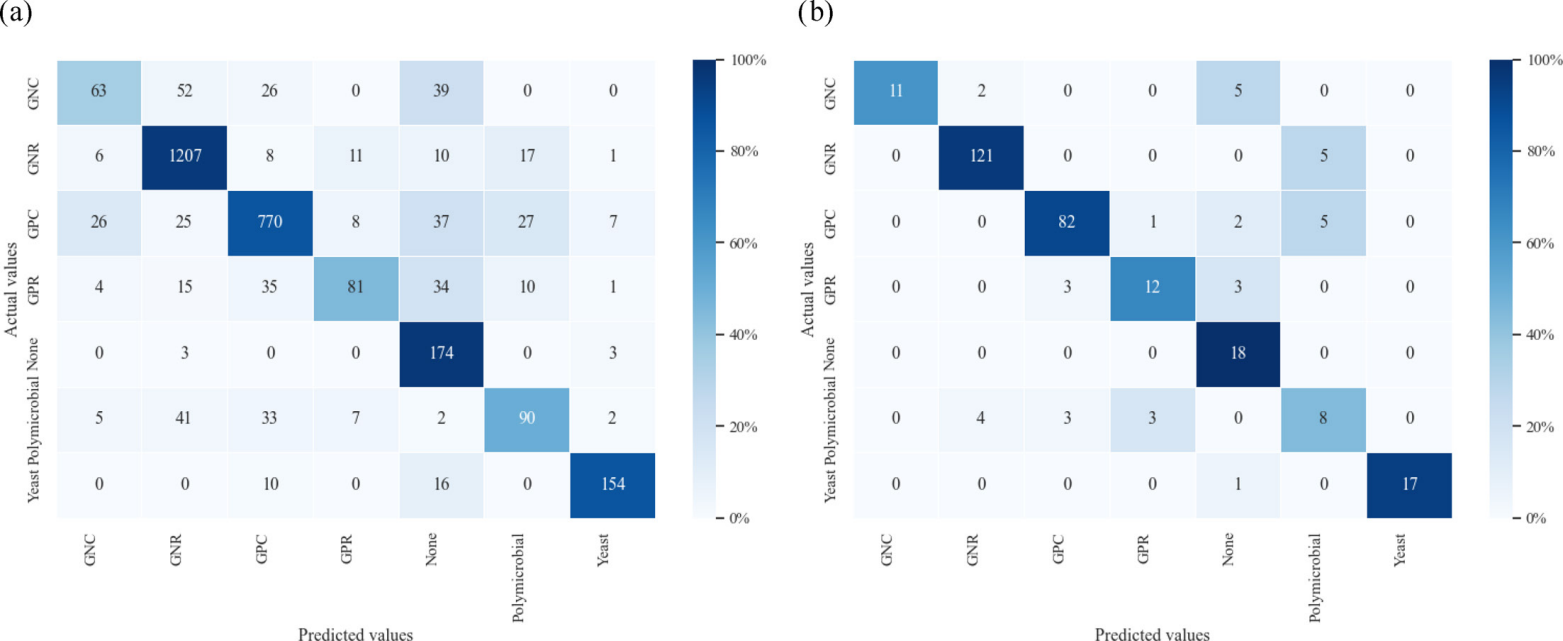
Confusion matrix for Class 1 predictions, classified by bacterial morphology. (**a**) MS. (**b**) CAD system. A heat map was created by deriving the proportion from the number of actual values as the denominator and the number of correct predictions as the numerator. GNC, Gram-negative cocci; GNR, Gram-negative rod; GPC, Gram-positive cocci; GPR, Gram-positive rod.

The overall accuracy for the 3,060 predictions by MS was 83.0% (95% CI: 81.6–84.3%), and the accuracy of results by individual MS ranged from 75.1% to 89.5% (standard deviation/mean: 0.048). The Fleiss’ kappa coefficient and AC1 statistic were 0.79 and 0.85 at NCGM and 0.68 and 0.77 at KUH, with substantial to almost perfect inter-rater agreement at both sites. The confusion matrix for the MS is shown in Fig. 3a. Predictions were relatively accurate for the GNR, yeast and none categories but were less accurate for classifying polymicrobial, GNC, GPC and GPR. The macro-average sensitivity of MS predictions was 79.1%. The results for other secondary outcomes are shown in Table 3. The overall accuracy for the 306 predictions by CAD was 87.9% (95% CI: 83.7–91.3%), with a macro-average sensitivity of 79.1%. The confusion matrix of CAD is shown in Fig. 3b. Predictions were relatively accurate for the GNR, GNC, GPC, yeast and none categories, but less accurate when classifying polymicrobial and GPR. The accuracy difference between the MS and CAD results was −4.93% (95% CI: −8.43% to −0.62%; *P*<0.001), indicating non-inferiority of interpretation by CAD compared with MS based on the Farrington and Manning analysis. Incorrect predictions were classified as cases of incorrect identification as rod or cocci (misprediction of morphology), incorrect identification as gram-positive or negative (misprediction of staining colour), both (misprediction of morphology and staining colour), incorrect identification of the presence of a microorganism, incorrect identification of the absence of a micro-organism and other unclassified prediction errors (Fig. S1). The percentage of morphological misprediction, staining misprediction, morphological and staining misprediction, incorrect identification of the presence of a microorganism, incorrect identification of the absence of a microorganism and unclassified prediction errors among MS was 3.9%, 2.5%, 1.2%, 0.2%, 4.5% and 4.6%, respectively (Fig. 3a). The percentage of morphological misprediction, staining misprediction, morphological and staining mispredictions, incorrect identification of the presence of a microorganism, incorrect identification of the absence of a microorganism and unclassified misprediction using CAD was 2.0%, 0%, 0%, 0%, 3.6% and 6.5%, respectively (Fig. 3b). In 2,424 predictions, both MS and CAD were correct but were incorrect in 255 predictions. Among the remaining images, 115 and 266 were correct only for MS and CAD, respectively (Table S5a). The agreement between correct and incorrect answers for MS and CAD was relatively moderate on Cohen’s kappa (0.50). However, the AC1 statistic showed an almost perfect agreement rate of 0.83. Because the prevalence index was high, the moderate kappa value appears to be due to a kappa paradox situation [16]. The agreement between MS and CAD is likely to be high because AC1 statistics are robust for this paradox.

**Table 3.** Precision, recall and F1 score (harmonic mean) for Class 1 (morphology)

	CAD			Microbiology specialist		
	Precision	Recall	F1	Precision	Recall	F1
None	62.1%	100.0%	76.6%	55.8%	96.7%	70.7%
Polymicrobial	44.4%	44.4%	44.4%	62.5%	50.0%	55.6%
GPC	93.2%	91.1%	92.1%	87.3%	85.6%	86.4%
GPR	75.0%	66.7%	70.6%	75.7%	45.0%	56.4%
GNR	95.3%	96.0%	95.7%	89.9%	95.8%	92.7%
GNC	100.0%	61.1%	75.9%	60.6%	35.0%	44.4%
Yeast	100.0%	94.4%	97.1%	91.7%	85.6%	88.5%

CAD, computer-aided diagnosis system; F1, harmonic mean of precision and recall; GNC, Gram-negative cocci; GNR, Gram-negative rod; GPC, Gram-positive cocci; GPR, Gram-positive rod.

### Class 2 (species)

**Fig. 4.**
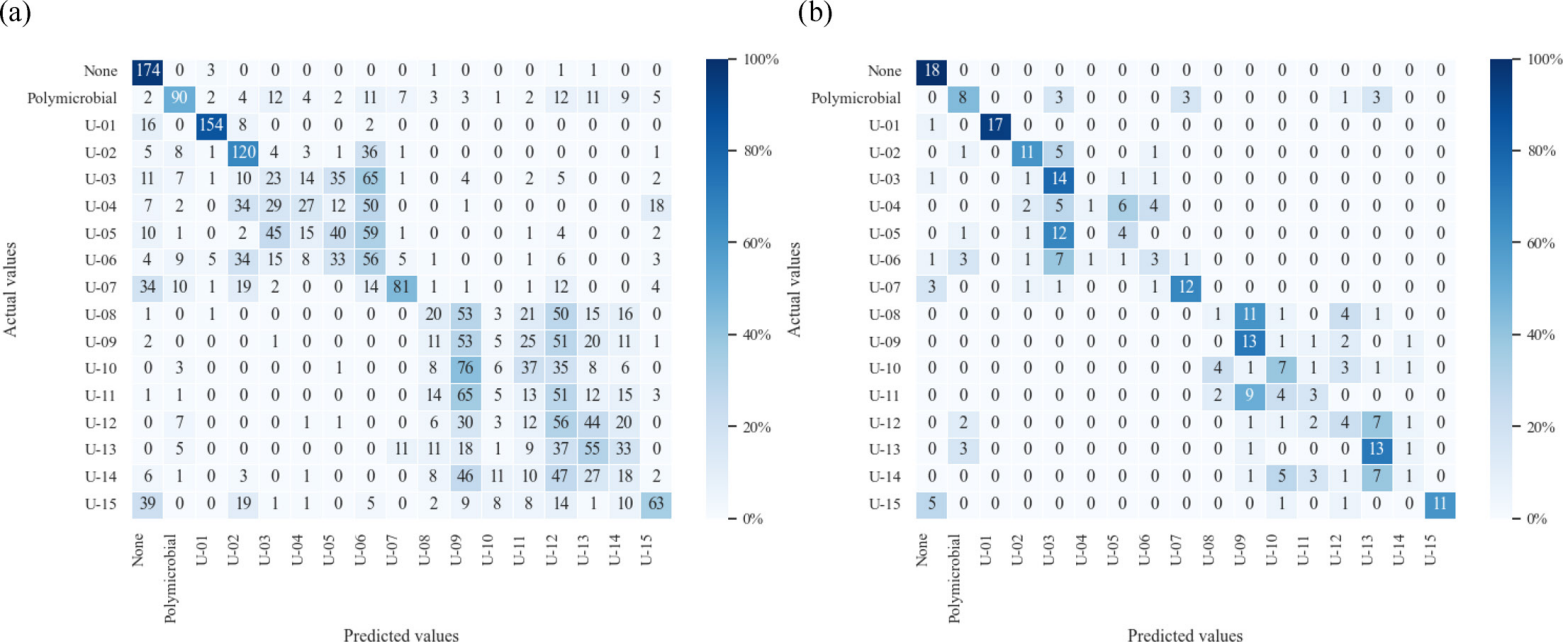
Confusion matrix for Class 2 predictions, classified by detailed bacterial species or groups. (**a**) MS. (**b**) CAD system. A heat map was created by deriving the proportion from the number of actual values as the denominator and the number of correct predictions as the numerator.

The accuracy for MS was 34.3% (95% CI: 32.6–36.0%), ranging from 24.2% to 46.4% for individuals (standard deviation/mean: 0.155). The Fleiss’ kappa coefficient and AC1 statistic of 0.40 and 0.44 for NCGM and 0.31 and 0.37 for KUH indicated fair to moderate inter-rater agreement. The confusion matrix for the MS is shown in Fig. 4a. The predictions were often incorrect when classifying GPC (‘U-02’–‘U-06’) and GNR (‘U-08’–‘U-14’). In particular, the precision and recall of GNR tended to be low, ranging from 9.2% to 28.4% and 3.3% to 31.1%, respectively (Table 4). The accuracy of CAD was 46.1% (40.4%–51.8%). As with MS, the predictions by CAD were often incorrect when classifying GNRs, whereas the predictions were more accurate for GPCs, especially in the GPC cluster (Fig. 4b, Table 4). The macro-average sensitivity of MS and CAD was 34.3% and 46.1%, respectively. The precision and recall of GNRs of CAD tended to be higher than those of MS, ranging from 14.3% to 40.6% and 5.6% to 72.2%, respectively (Table 4). In particular, the F1 scores for glucose non-fermenting bacteria were 38.4% for MS and 60.3% for CAD (Table 5).

**Table 4.** Precision, recall and F1 value (harmonic mean) for Class 2 (species)

		CAD			Microbiology specialist		
		Precision	Recall	F1	Precision	Recall	F1
U-01	*Candidaspp*.	100.0%	94.4%	97.1%	91.7%	85.6%	88.5%
U-02	GPC cluster	64.7%	61.1%	62.9%	47.4%	66.7%	55.4%
U-03	*Enterococcus faecalis*	29.8%	77.8%	43.1%	17.4%	12.8%	14.7%
U-04	*Enterococcus faecium*	50.0%	5.6%	10.0%	36.5%	15.0%	21.3%
U-05	*Streptococcus agalactiae*	33.3%	22.2%	26.7%	32.0%	22.2%	26.2%
U-06	Other GPC	30.0%	16.7%	21.4%	18.8%	31.1%	23.4%
U-07	*Corynebacteriumspp*.	75.0%	66.7%	70.6%	75.7%	45.0%	56.4%
U-08	*Enterobacter cloacae*	14.3%	5.6%	8.0%	23.3%	11.1%	15.0%
U-09	*Escherichia coli*	35.1%	72.2%	47.3%	14.8%	29.4%	19.7%
U-10	*Klebsiella oxytoca*	35.0%	38.9%	36.8%	14.0%	3.3%	5.4%
U-11	*Klebsiella pneumoniae*	30.0%	16.7%	21.4%	9.2%	7.2%	8.1%
U-12	Other GNR *Enterobacteriaceae*	25.0%	22.2%	23.5%	14.7%	31.1%	20.0%
U-13	*Pseudomonas aeruginosa*	40.6%	72.2%	52.0%	28.4%	30.6%	29.4%
U-14	Other GNR glucose non-fermenting bacteria	20.0%	5.6%	8.7%	13.0%	10.0%	11.3%
U-15	GNC	100.0%	61.1%	75.9%	60.6%	35.0%	44.4%
Poly	Polymicrobial	44.4%	44.4%	44.4%	62.5%	50.0%	55.6%
None	None	62.1%	100.0%	76.6%	55.8%	96.7%	70.7%

CAD, computer-aided diagnosis system; F1, harmonic mean of precision and recall; GNC, Gram-negative cocci; GNR, Gram-negative rod; GPC, Gram-positive cocci; GPR, Gram-positive rod.

**Table 5.** Classification of Gram-negative rods

	CAD						Microbiology specialists*					
	GF	NF	Others	Precision	Recall	F1	GF	NF	Others	Precision	Recall	F1
GF	76	12	2	75.2%	84.4%	79.6%	709	167	24	78.8%	70.1%	74.2%
NF	11	22	3	59.5%	61.1%	60.3%	198	133	29	36.9%	40.1%	38.4%
Others	14	3	163				104	32	1664			

a *The results are the combined results of interpretations by 10ten microbiology specialists.

CAD, computer-aided diagnosis system; F1, harmonic mean of precision and recall; GF, glucose fermenters; NF, glucose non-fermenters.

The accuracy difference was –11.8% (95% CI: –17.6% to –6.04%; *P*<0.001), indicating non-inferiority of interpretation by CAD compared with MS, based on the Farrington and Manning analysis. However, the 95% CI indicated that the CAD interpretation was more accurate than the MS interpretation.

Both interpretations by MS and CAD were correct for 749 images but incorrect for 1,350 images. Among the remaining images, 300 and 661 were correct only for MS and CAD, respectively (Table S5b). Compared with Class 1, the agreement on answers was lower for MS and CAD, with a Cohen’s kappa coefficient of 0.36 (fair) and an AC1 statistic of 0.43 (moderate).

## Discussion

The results of this study suggest that CAD is non-inferior to MS for interpreting Gram-stain results in urine specimens. Although there have been attempts to use image AI analysis based on deep-learning methods to assess Gram-stain results with blood cultures [17] and cervical smears in bacterial vaginosis [18], to our knowledge, this is the first study to compare and validate Gram-stain results interpreted by CAD and MS using clinical urine specimens. Based on the F1 scores, in Class 1 (morphology) differentiation, CAD performed better than MS with GPR and GNC but less well with polymicrobial infections. The accuracy of the Gram-stain readings by MS was 83%. The Gram-staining error rate in discriminating bacterial morphologic features, verified in multiple institutions, is approximately 5% [19]. Microbiologists in Japan are assessed annually by the Japanese Association of Medical Technologists to check the quality of their Gram-stain interpretation (data not shown), with the error rate generally less than 5%. However, MS performance in this study was poor relative to the standard error rate, notwithstanding an accuracy in discriminating the five microbial classifications (GPC, GPR, GNR, GNC and yeast) in urine specimens by 11 MS of 96.4% in a pilot study [12]. Although the pilot study findings were validated for all categories except the polymicrobial and none categories, the interpretation of slides with polymicrobial infections by both MS and CAD was poor in this study. MS also displayed poor accuracy in interpreting slides with GPR. However, compared with GNR and GPC, GPRs (*Corynebacterium spp*.) are rare in clinical urine specimens. In this study, because samples were not selected to reflect their epidemiology, it is possible that the overrepresentation of GPR relative to their prevalence in real-world clinical settings affected the overall accuracy of the results and may have contributed to the high error rate of classification of MS. Although classified as GPR, *Corynebacterium spp*. have heterogeneous morphology and may have a coccoid shape, depending on the culture conditions [20,21]. In this study, *Corynebacterium spp*. accounted for less than 1% of the eligible samples, but they accounted for about 6% of the datasets in this study, which was a higher proportion than in the real world. Although this was not the only reason for the high error rate, this might have contributed to the high error rate.

The effect of accurate Gram-stain interpretation on antimicrobial use is not clear; however, Gram-stain results influence empirical antibiotic selection for common infectious diseases such as community-acquired pneumonia [22]. A randomized controlled trial on ventilator-associated pneumonia showed that selection by guideline compliance reduced broad-spectrum antimicrobial use, with no change in clinical effectiveness [23]. Using either PCGS or guidelines in patients with infectious diseases may lead to the selection of targeted antibiotic treatment in patients with UTIs [8]. In paediatric patients with UTI, PCGS showed high diagnostic performance and concordance with bacteria detected in culture, suggesting that PCGS contributes to more efficient selection of appropriate antibiotics [24]. Taniguchi *et al*. [25] noted that PCGS selection of antimicrobial agents based on PCGS results and information such as antibiograms resulted in the selection of antimicrobials matching the susceptibility of many detected uropathogens in adult patients with UTI. Although the predictive value of antibiograms for appropriate empirical antimicrobials may not be high [26], antibiograms are one of the few pieces of information available for empirical treatment selection, and their use may contribute to reducing broad-spectrum antibiotic use.

As with the eligible specimens shown in Table S2, the main UTI pathogens are GNR [27]. In this study, the F1 value for GNR was more than 95% for CAD, which is higher than that of MS. Therefore, CAD satisfies the prerequisites for estimating the causative organism of UTI. Differentiating bacterial species by Gram staining has shown differentiation between *Staphylococcus aureus* and other staphylococci in blood culture [28,30] and between penicillin-resistant and penicillin-sensitive enterococci in blood culture [31]. Furthermore, the length and thickness ratio of GNR can differentiate between fermenting and non-fermenting bacteria [32]. Although these studies showed relatively good performance, achieving sufficient accuracy when differentiating among species rather than within species remains challenging, as shown in this study. However, in this study, the Class 2 performance by MS was not high. Although the differential performance of the GPC cluster was relatively good, the precision and recall of the main pathogens such as GNRs tended to be lower for MS than for CAD (Tables3  4). The F1 score of non-fermenting bacteria was <40% in MS and >60% in CAD. Although the interpretation by both CAD and MS in this study was suboptimal, in clinical practice, PCGS interpretation by medical professionals including MS has reduced the use of broad-spectrum antimicrobial agents compared with guideline-based antimicrobial use [8]. Therefore, further clinical evaluation is needed to provide a better understanding of the true effectiveness of PCGS using CAD for guiding clinicians in their choice of empiric antimicrobial therapy; for example, if the bacteria seen in the Gram stain are only GPC, ampicillin can be selected by taking *Enterococcus faecalis* into account, and if *E. coli* or *Pseudomonas aeruginosa* can be assumed based on the presence of GNR, it can be optimized for antimicrobial agents, under real-world conditions.

This study has some limitations. First, it was conducted in two tertiary centres. Although the B and M method was used in both centres, the reagents used for Gram staining, staining technique, cleanliness of the microscope and pre-treatment may differ between centres. These factors may affect the interpretation of CAD, limiting the generalizability of the results. Second, the included samples did not reflect the distribution of pathogens causing UTI in clinical practice. However, it is possible to generalize the results to bacterial species, and the samples included the most frequently detected species causing UTIs detected in a previous single-centre study [27] and national surveillance in Japan [33]. Third, samples in which the bacteria detected by microscopy and those detected by culture differed were excluded. As such samples can be found in real-world clinical practice, ideally CAD should be able to classify samples with discrepant microscopy and culture results. However, a limited number of samples were excluded based on discrepant microscopy and culture results. When bacteria are not observed on microscopy, the bacterial count is likely to be low, even if there is growth on culture [34]. This suggests that the sample may be contaminated and that the bacteria grown on culture may be contaminants. To confirm clinical effectiveness, it is necessary to use samples that reflect the spectrum of bacteria in clinical practice. Fourth, this CAD model did not consider whether the patient had received prior antimicrobial therapy. Owing to the possibility of changes in bacterial morphology such as elongation and bulging due to prior antimicrobial therapy, we performed a pilot validation before developing the AI model (data not shown) to assess whether the accuracy of discrimination differed according to whether the patient had received prior antimicrobial therapy. However, there was no significant difference in the accuracy of the classification, and because few patients had received prior antimicrobial therapy, it was not logistically feasible to collect and analyse the training data for various types of bacteria separately according to whether or not the patient had received prior antimicrobial therapy. Therefore, we decided not to collect information on prior use of antimicrobial agents. Lastly, because the AI model was constructed using images of stained B and M microscopic images taken with an iPhone, it remains unclear whether CAD can be generalized to Gram-staining methods other than the B and M method and imaging data captured by smartphone devices other than the iPhone. Further studies using a range of methods to create the training data are needed to resolve these questions.

CAD interpretation of Gram-stain results in urine specimens following B and M methods for staining samples using images captured by an iPhone camera is non-inferior to interpretation by MS. Although CAD may be able to facilitate infectious disease treatment using PCGS for urine when MS is unavailable to interpret Gram-stain results, future studies are needed to determine the generalizability of these results.

## Supplementary material

10.1099/jmm.0.002008 Uncited Supplementary Material 1.

## Abbreviations

AC1: first-order agreement coefficient
ATCC: American Type Culture Collection
B and M: Bartholomew and Mittwe
CAD: computer-aided diagnosis
CI: confidence interval
GNC: Gram-negative cocci
GNR: Gram-negative rod
GPC: Gram-positive cocci
GPR: Gram-positive rod
KUH: Kobe University Hospital
MS: microbiology specialists
NCGM: National Center for Global Health and Medicine
PCGS: point-of-care Gram staining
UTIs: urinary tract infections
