# Supplementary material for: Accuracy of classification of urinary Gram-stain findings by a computer-aided diagnosis app compared with microbiology specialists
Source: J Med Microbiol. 2025 Apr 23;74(4):002008. doi: 10.1099/jmm.0.002008 (PMC12018707; doi:10.1099/jmm.0.002008)
Supplement: Uncited Supplementary Material 1. [file jmm-74-02008-s001.pdf]

Supplementary materials

Accuracy of classification of urinary Gram-stain findings by a computer-aided diagnosis app compared with microbiology specialists

Kei Yamamoto [a], Goh Ohji [b,c], Isao Miyatsuka [d], Kei Furui-Ebisawa [b], Ataru Moriya [e,f], Shogo Maeta [d], Hidetoshi Nomoto [a], Masami Kurokawa [e], Kenichiro Ohnuma [c], Mari Kusuki [c], Yukari Uemura [g], Norio Ohmagari [a]

[a] Disease Control and Prevention Centre, National Centre for Global Health and Medicine, Tokyo, Japan

[b] Division of Infectious Diseases Therapeutics, Department of Microbiology and Infectious Diseases, Kobe University Graduate School of Medicine, Kobe, Japan

[c] Department of Clinical Laboratory, Kobe University Hospital, Kobe Japan

[d] CarbGeM Inc., Tokyo, Japan

[e] Department of Clinical Laboratory, National Centre for Global Health and Medicine, Tokyo, Japan

[f] Department of Clinical Laboratory, Ibaraki Higashi National Hospital

[g] Centre for Clinical Sciences, National Centre for Global Health and Medicine, Tokyo, Japan

Corresponding Author: Kei Yamamoto

Disease Control and Prevention Centre, National Centre for Global Health and Medicine

1-21-1 Toyama, Shinjuku-ku, Tokyo 162-8655, Japan

Phone: +81-3-3202-7181

Fax: +81-3-3202-1012

E-mail: kyamamoto@hosp.ncgm.go.jp

Supplementary Appendix S1. Artificial intelligence (AI) model building

To prevent the learning of slide-by-slide features rather than bacterial features, data splitting was performed on a per-slide basis rather than on the entire image. This ensured that images derived from the same slide did not cross over into the training, validation, or test datasets.

We created three types of models: a model that classifies seven categories of morphology including 1) yeast (*Candida* spp.), 2) Gram-positive cocci (GPC), 3) Gram-positive rods (GPR), 4) Gram-negative rods (GNR), 5) Gram-negative cocci (GNC), 6) non-fermenting bacterial class, and 7) multiple bacterial class (Class 1 model). A model that classifies the bacterial species as GPC in the Class 1 model (GPC model) and another that classifies them as GNR in the Class 1 model (GNR model). We created the Class 1 model as a multi-label classification model and the GPC and GNR models as multi-class image classification models.

We used a fine turning technique based on the ConvNeXt [1] convolutional neural network (CNN) architecture pre-trained on the ImageNet Large Scale Visual Recognition Competition (ILSVRC) 2012 image database [2].

We used 20% of all slides as the test dataset and divided the remaining 80% into four parts. We performed a four-fold cross-validation (4-fold CV) by using three of the four parts as the training dataset (training) and the remaining part as the validation dataset. We then built a model. Because the dataset contained only one clinical specimen for GNC, the images of the clinical specimen were divided in the same proportion as above to construct the dataset.

The batch size was set to 32 because we used Gradient Accumulation with a mini-batch size of 2 and 16 gradient accumulations.

To evaluate the loss function, we used Multi-Label Evidential Loss [3] for the Class 1 model, and Evidential Loss [4] for the GPC and GNR models, and used AdamW [5] as an optimizer. Data augmentation was performed using the RandAugment [6] module in PyTorch.

For the construction of the Class 1 model, we also used an augmentation method called mixup [7], which combines multiple images.

We used accuracy, recall, precision, and f_β (β=0.5) as evaluation metrics. We monitored recall on the validation data and adopted the model that achieved the highest score. The f_β score was

based on the following equation:

$$f_\beta = (1+\beta^2)\frac{precision \cdot recall}{\beta^2 \cdot precision + recall}$$

We built a model by optimizing the weights using the Model Soups [8] method on the models constructed by the 4-fold CV.

Model evaluation

During inference, the image was resized to 1024 × 1024 after center cropping of 3024 × 3024 and input to the Class 1 model. If the output of the Class 1 model was GNR or GPC, the corresponding model is used to classify the species, and the Class 1 category and classification results were output. If EvidentialLoss was used as the loss function, the uncertainty u and confidence conf were calculated as follows:

$$f(x) = \begin{cases} x\,(x>0) \\ 0\,(x\le 0) \end{cases}$$

$$u = \frac{K}{\sum_{k=0}^{K}\left(f_k(x)+1\right)}$$

$$conf = 1 - u$$

where K is the number of classification classes.

When MultilabelEvidentialLoss was used as the loss function, the uncertainty was calculated for each class according to the following equation using two outputs: present (+) and absent (−) for each class.

$$u_k = \frac{2}{¿¿}$$

The maximum value was the output from the following equation, as uncertainty for the image:

$$u = max\left(\{u_k\}_{k=1}^{K}\right)$$

In addition to the confidence intervals, probabilities were calculated using a sigmoid function

for the Class 1 model and a softmax function for the GNR and GPC models.

References

1. Liu Z, Mao H, Wu CY, Feichtenhofer C, Darrell T, Xie S. A ConvNet for the 2020s. In: Proceedings of the IEEE/CVF conference on computer vision and pattern recognition (CVPR); 2022:11966-76. doi:10.1109/CVPR52688.2022.01167.

2. Russakovsky O, Deng J, Su H, Krause J, Satheesh S, Ma S, et al. ImageNet large scale visual recognition challenge. Int J Comput Vis. 2015;115(3):211-52. doi:10.1007/s11263-015-0816-y.

3. Zhao C, Hu C, Shao H, Wang Z, Wang Y 2022. Towards trustworthy multi-label sewer defect classification via evidential deep learning. arxiv https://arxiv.org/abs/2210.13782. doi:10.1109/ICASSP49357.2023.10096569.

4. Sensoy M, Kaplan L, Kandemir M. Evidential deep learning to quantify classification uncertainty. arxiv https://arxiv.org/abs/1806.01768. 2019;2018.

5. Loshchilov I, Hutter F. Decoupled weight decay regularization. [arxiv https://arxiv.org/abs/1711.05101].

6. Cubuk ED, Zoph B, Shlens J, Le QV 2019. RandAugment: practical automated data augmentation with a reduced search space. arxiv https://arxiv.org/abs/1909.13719. doi:10.1109/CVPRW50498.2020.00359.

7. Zhang H, Cisse M, Dauphin YN, Lopez-Paz D 2018. mixup: Beyond empirical risk minimization. [arxiv https://arxiv.org/abs/1710.09412?ref=inference.vc].

8. Wortsman M, Ilharco G, Gadre SY, et al. 2022. Model soups: averaging weights of multiple fine-tuned models improves accuracy without increasing inference time. arxiv https://arxiv.org/abs/2203.05482.

Supplementary Table S1: Samples used as training data for building the artificial intelligence model

Microorganism	Slides from NCGM	Slides from KUH	Total
Polymicrobial	144	18	162
None	46	14	60
Candida spp.	103	33	136
GPC cluster	63	22	85
Enterococcus faecalis	37	30	67
Enterococcus faecium	19	20	39
Streptococcus agalactiae	22	21	43
Other GPC	15	20	35
Corynebacterium spp.	32	38	70
Enterobacter cloacae	16	27	43
Escherichia coli	235	56	291
Klebsiella oxytoca	18	28	46
Klebsiella pneumoniae	65	27	92
Other GNR *Enterobacteriaceae*	65	20	85
Pseudomonas aeruginosa	32	15	47
Other GNR glucose non-fermenting bacteria	5	22	27
GNC	7	10	17
*Others	5	-	5
Total	929	421	1350

GNC, Gram-negative cocci; GNR, Gram-negative rod; GPC, Gram-positive cocci; KUH, Kobe University Hospital; NCGM, National Center for Global Health and Medicine.

* Others include slides on which two types of GNR or GPC bacteria were detected were classified as others. They were used for modeling as GNR (1 slide) and GPC (4 slides) for Class 1 classification, but not for Class 2 classification.

Supplementary Appendix S2. Culture and preparation method for spiked samples

(1) The stored strain was incubated on a CO_2-rich chocolate agar medium for 24 h.

(2) The isolating strain was adjusted to a concentration of McFarland 0.5 and further diluted 100-fold.

(3) A total of 3 mL of medium supplemented with cation-adjusted Muller-Hinton Bouillon and horse hemolytic blood 2.5–5% was inoculated with 10 µL of the adjusted solution in described in (2) above.

(4) After incubation in CO_2 rich medium overnight (approximately 16–18 h), the culture medium was suspended in bacteria-free urine or saline solution of the same pH as the urine, and the solution was applied to a glass slide for Gram staining.

Supplementary Table S2. Eligible samples

	NCGM	KUH
Candida spp.	29 (3/0)	13 (3/0)
GPC cluster	31 (3/0)	18 (3/0)
Enterococcus faecalis	18 (3/0)	9 (3/0)
Enterococcus faecium	5 (3/0)	1 (1/2)
Streptococcus agalactiae	11 (3/0)	3 (3/0)
Other GPC	4 (3/0)	2 (2/1)
Corynebacterium spp.	14 (3/0)	5 (3/0)
Enterobacter cloacae	6 (3/0)	3 (3/0)
Escherichia coli	87 (3/0)	26 (3/0)
Klebsiella oxytoca	7 (3/0)	1 (1/2)
Klebsiella pneumoniae	17 (3/0)	13 (3/0)
Other GNR *Enterobacteriaceae*	13 (3/0)	11 (3/0)
Pseudomonas aeruginosa	16 (3/0)	5 (3/0)
Other GNR glucose non-fermenting bacteria	0 (0/3)	0 (0/3)
GNC	0 (0/3)	0 (0/3)
Polymicrobial	292 (3/0)	419 (3/0)
None	838 (3/0)	1,340 (3/0)
Total	1,388 (45/6)	1,869 (40/11)

The number in parentheses indicates the number of samples used in the dataset, and is listed as "clinical samples/spiked samples".

GNC, Gram-negative cocci; GNR, Gram-negative rod; GPC, Gram-positive cocci; KUH, Kobe University Hospital; NCGM, National Center for Global Health and Medicine.

Supplementary Table S3. The morphological findings of the bacteria in the excluded samples

	Deviation from the results of the culture		Multiple bacteria culture		Bacteria not included in the classification	
	NCGM	KUH	NCGM	KUH	NCGM	KUH
GPC	7	21	68	25	0	0
GPR	36	104	39	11	7	0
GNC	0	0	1	0	0	0
GNR	4	6	34	59	2	0
Yeast	0	0	13	15	0	0

GNC, Gram-negative cocci; GNR, Gram-negative rod; GPC, Gram-positive cocci; GPR, Gram-positive rod; KUH, Kobe University Hospital; NCGM, National Center for Global Health and Medicine.

Supplementary Table S4. ATCC standard strains used in the spiked samples

NCGM	KUH
Acinetobacter lwoffii ATCC17925	*Enterococcus faecium* ATCC27270
Acinetobacter species ATCC49139	*Enterococcus faecium* ATCC35667
Stenotrophomonas maltophilia ATCC13637	*Aerococcus viridans* ATCC10400
Neisseria gonorrhoeae ATCC31426	*Klebsiella oxytoca* ATCC49131
Neisseria gonorrhoeae ATCC19424	*Klebsiella oxytoca* ATCC8724
Neisseria gonorrhoeae ATCC43070	*Alcaligenes faecalis* ATCC8750
	Stenotrophomonas maltophilia ATCC17666
	Stenotrophomonas maltophilia ATCC51331
	Neisseria gonorrhoeae ATCC19424
	Neisseria meningitidis ATCC13077
	Neisseria gonorrhoeae ATCC31426

KUH, Kobe University Hospital; NCGM, National Center for Global Health and Medicine

Supplementary Figure S1. Classification of misprediction corresponding to the confusion matrix in Figure 3

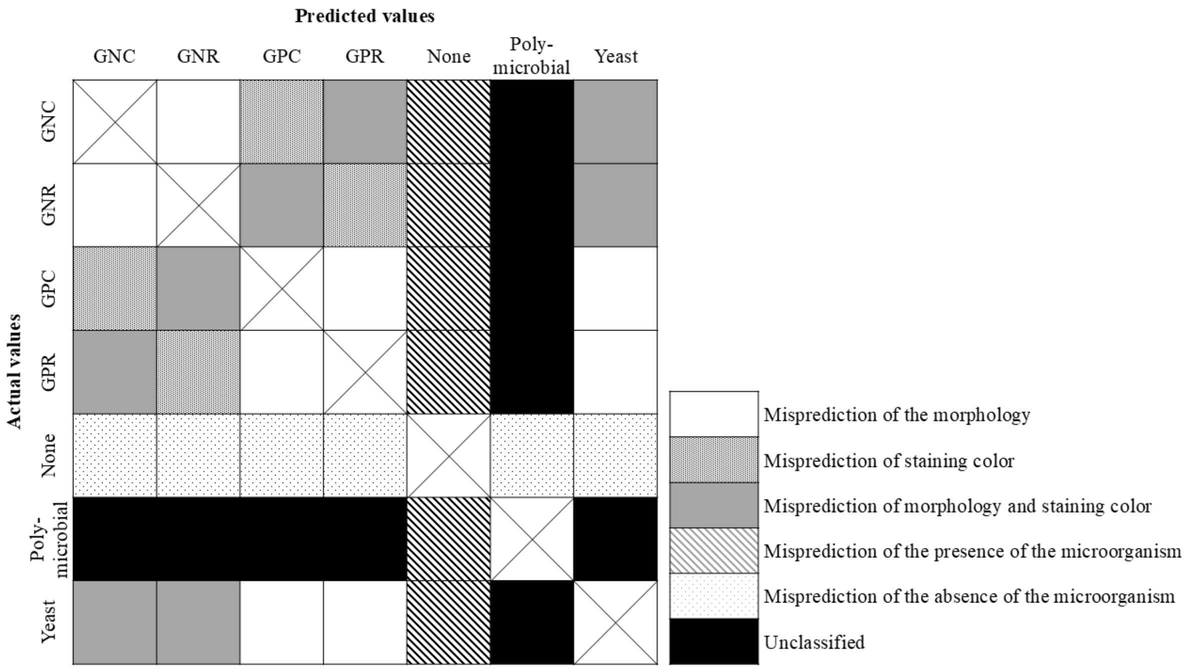

Supplementary Table S5. Agreement between correct and incorrect answers between microbiology specialists and computer-aided diagnosis systems

	MS correct	MS incorrect
**a) Class 1**		
CAD correct	2424	266
CAD incorrect	115	255
**b) Class 2**		
CAD correct	749	661
CAD incorrect	300	1350
